# Factors influencing pregnancy outcome with special attention to modified slow-release insemination and a patient-centred approach in a donor insemination programme: a prospective cohort study

**DOI:** 10.52054/FVVO.14.2.027

**Published:** 2022-07-01

**Authors:** W Ombelet, I Van der Auwera, H Bijnens, C Kremer, L Bruckers, G Mestdagh, N Dhont, R Campo

**Affiliations:** Genk Institute for Fertility Technology, Department of Obstetrics and Gynaecology, Schiepse Bos 6, 3600 Genk, Belgium; Faculty of Medicine and Life Sciences, Hasselt University, Martelarenlaan 42, Hasselt, Belgium; Interuniversity Institute for Biostatistics and statistical Bioinformatics, Data Science Institute, Hasselt University, Martelarenlaan 42, Hasselt, Belgium

**Keywords:** clinical pregnancy rate, donor sperm, infertility, intrauterine insemination, patient-centred care, slow-release insemination

## Abstract

**Introduction:**

A higher pregnancy rate after slow-release insemination instead of bolus injection was described in previous studies. Besides an effective medical treatment most patients wish to receive a patient-centred approach with sufficient emotional support.

**Study question:**

Does a patient-friendly approach with slow-release insemination (SRI) increase the clinical pregnancy rate (CPR) after intrauterine insemination (IUI) with donor semen?

**Study design, size, duration:**

The data of an ongoing prospective cohort study were analysed investigating the results of 1995 donor inseminations in 606 women from July 2011 until December 2018. As from January 2016 the insemination procedure was performed by midwives instead of medical doctors. Instead of bolus injection of sperm a slow-release IUI was done together with a more patient-centred approach.

**Materials and methods:**

The data of 1995 donor inseminations were analysed to study the importance of different covariates influencing IUI success. Generalized estimating equations (GEEs) were used for statistical analysis. Results of two periods (2011-2015 and 2016-2018) were examined and compared.

**Results:**

Clinical pregnancy rates (with foetal heartbeat) following donor inseminations increased from 16.6 % to 20.8 % per cycle, a non-significant increase (p=0.061).

**Conclusion:**

A more patient-friendly approach with slow-release of processed semen resulted in a non-significant higher clinical pregnancy rate of 4.2 % per cycle after donor insemination.

## Introduction

IUI with donor semen (IUI-D) is indicated for couples suffering from severe male factor infertility, including azoospermia, not preferring IVF-ICSI as a possible option. IUI-D is also indicated for men with genetic disorders linked to the Y chromosome that might be transmissible to the progeny. It is increasingly used for lesbians and women without a male partner (singles).

In Belgium the demand for IUI-D has increased during the last decade due to the rise in the number of lesbian couples and single women seeking infertility care and a high inflow of patients searching for cross-border reproductive care to avoid restrictive laws in their home country (Thijssen et al., 2014; Pennings, 2004; Pennings et al., 2009; Van Hoof et al., 2015)

According to the 2017-2018 BELRAP data (Belgian Register for Assisted Procreation, www.belrap.be) the Clinical Pregnancy Rate (CPR) and Live Birth Rate (LBR) per IUI attempt with donor semen was respectively 13.9 % (2227/16005) and 12.5 % (1927/15428) and according to the results generated from European registries by ESHRE the delivery rate for donor inseminations was 12.4 % (6249/50467) in 2016, with a 8.1 % multiple pregnancy rate (Wyns et al., 2020).

Compared to IVF these figures seem to be disappointing, therefore different strategies have been investigated to improve the delivery rate per IUI cycle.

A better selection of women who are the best candidates for IUI is mandatory. For example, a significant negative effect of human papilloma virus (HPV) positivity in men and/or women on clinical pregnancy rates following IUI has been reported (Depuydt et al., 2016; 2019). Depuydt et al. (2018) showed that when HPV positive donor sperm was used, no clinical pregnancies resulted, whereas if HPV negative donor sperm was used the clinical pregnancy rate was 14.6%. From both a cost/ benefit and a safety point of view it is recommended that donor sperm should always be tested for HPV before using it for insemination.

Secondly, we can try to increase success rates by changing the technique and methodology of IUI per cycle.

Previous reports showed that a slow-release intrauterine insemination might improve the pregnancy rate compared to bolus IUI. In these studies, a commercially available product was used (Grasby auto-syringe driver or the EVIE Slow- Release Insemination Pump) (Muharib et al., 1992; Marschalek et al., 2017, 2020).

We recently published the data of the IUI outcome results using homologous semen in 2 different periods. In the first period a bolus IUI was carried out, in the second period a modified slow- release IUI combined with a more patient-centred approach was performed. It turned out that the CPR increased significantly from 9.0 % to 13.5 % in the second period (p = 0.0016) (Ombelet et al., 2021). For that specific study and the present one we made use of the registered data of an ongoing prospective cohort study in the ZOL Hospitals in Genk, a tertiary referral infertility centre (Thijssen et al., 2017a, 2017b). In this study we prospectively studied the CPR after donor insemination comparing a bolus injection and a modified slow insemination approach in two different periods.

**Figure 1:**
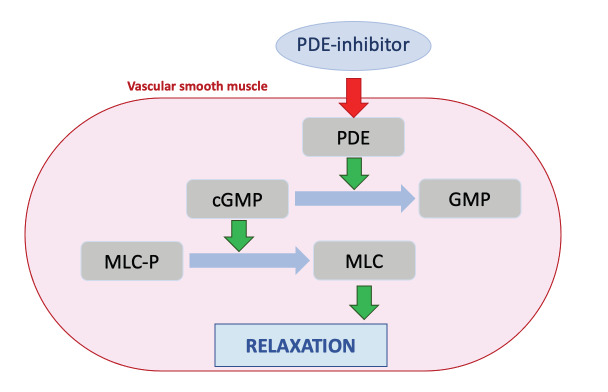
Simplified pathway of PDE. PDE = phosphodiesterase, (c)GMP = (cyclic) guanosine monophosphate, MLC(-P) = myosin light chain (-phosphate).

## Materials and Methods

Our data are part of a prospective observational cohort study performed at the Genk Institute for Fertility Technology (Thijssen et al, 2017a, 2017b). We studied the medical records of 606 patients undergoing 1995 inseminations with donor semen. All inseminations were performed between 1 July 2011 and 31 December 2018.

The sperm examination procedure, the semen processing techniques, and the conditions why IUI was performed in a natural cycle or after ovarian stimulation have been described before (Thijssen et al., 2017a, 2017b).

Until December 2015 (Period 1) the inseminations were performed by medical doctors (mostly trainees). Most of the time the patients had never seen the medical doctor before. A bolus injection of washed sperm was given (max 5-10 seconds). As from January 2016 (Period 2) and due to an increasing workload of the MDs it was decided that our midwives working in our infertility centre should perform the IUI procedure. It was perceived that the IUI performed by a midwife familiar with the patient and willing to discuss any concerns, would constitute a more patient friendly approach. During a short training course, the 5 midwives were trained to perform a slow-release sperm injection of at least 45-60 seconds, (Ombelet et al., 2021).

Patient selection and study design remained the same during the two investigated periods and have been described before (Thijssen et al., 2017b). Intrauterine inseminations were planned at 20–30 h post-HCG. A fraction of the washed motile spermatozoa was inserted up to the uterine fundus and expelled into the uterine cavity, bolus injection (by MDs) in period 1 (until December 2015) and modified slow-release IUI (by midwives) from January 2016 on (period 2). The women remained supine for 15 min while the midwives gathered information about a number of factors possibly influencing IUI outcome by means of a questionnaire. Comparing the data and outcome results of the 2 study periods (period 1: July 2011 - December 2015, period 2: January 2016 - December 2018), the only variables that changed were the duration of sperm injection (bolus versus slow-release) and the person who performed the IUI (MD versus midwife). Patient selection, cycle monitoring and use of natural cycle or ovarian stimulation remained the same in both periods.

Donor insemination was performed in 383 women during 1260 cycles in period 1 and in 223 women during 735 cycles in period 2.

Serum ß-HCG was determined 14–16 days after IUI. Clinical pregnancy rate (CPR) was defined as the ultrasound visualization of a gestational sac with foetal heartbeat at 7-8 weeks of gestation.

Ethical approval for this study was obtained on 31 May 2011 (reference number: 13/054U).

## Donor insemination: patient selection and studied covariates

All women were either single, lesbian, or heterosexual couples with an azoospermic partner or a partner with a y-linked chromosome genetic disorder. In all patients, a complete infertility work-up was carried out including a medical history, physical examination, pelvic ultrasound, serum hormone assays between day 2 and 4 of the menstrual cycle, and mid-luteal serum progesterone in women with regular menstrual cycles. If an implantation abnormality or uterine abnormality was suspected on ultrasound, a hysteroscopy, laparoscopy, or both, was carried out. Tubal patency was assessed either by HSG (hysterosalpingography), HyFoSy (hysterosalpingo-foam sonography) or laparoscopy. Biochemical and ultrasound monitoring was conducted in all treatment cycles.

## Statistical analysis

An important assumption of all classical statistical analyses, such as ordinary logistic regression, is the assumption of independency. In this study this assumption is not fulfilled. When a first IUI attempt failed, the patient probably will return for another attempt. Even if the patient becomes pregnant, they often will return to be treated again. This shows that not all observations are independent: some observations come from the same patient, whereas other observations come from different patients. Considering this dependency, we modelled the probability of becoming pregnant by means of a generalized estimating equations (GEE) model (Zeger and Liang, 1986; Molenberghs and Verbeke, 2005). This model is an extension of ordinary logistic regression, where the correlation between observations from the same person is taken into account.

Statistical significance was established at p < 0.05. All GEE analyses were done in the software package SAS® version 9.4 for Windows (Belgium). Continuous data are presented as mean ± standard deviation (SD), whereas categorical (or categorized) data are presented in terms of the CPR ± standard error (SE). First, a univariate analysis was performed to investigate the influence of several covariates on the CPR including female age (years), smoking (yes or no), BMI both (kg/m2), primary/ secondary infertility, cycle rank, ovarian stimulation method [natural cycle (NC), clomiphene citrate (CC), human menopausal gonadotrophin (HMG)/ recombinant FSH (rFSH)], Day 0 oestradiol (ng/l) and progesterone (µg/l) levels, human chorionic gonadotrophin (HCG)-insemination time interval (hours), easy or difficult insemination and sperm quality parameters [concentration (million/ml), motility grade A (%), progressive motility (%) and Insemination Motile Count after processing (IMC, million)].

The main characteristics of the study population before (period 1) and after (period 2) 2016 were compared using univariate GEE models.

Univariate GEE models were also used to assess the association between each covariate and CPR. All covariates that were associated with CPR in these univariate analyses (based on p < 0.20) were included in a multivariable GEE model. Backward model selection was performed, i.e., in each step the least significant (with p > 0.05) covariate was excluded from the model until only significant (p < 0.05) covariates remained. The covariate comparing both periods was not included in this model selection procedure but was added to the final model and kept only if significant (p < 0.05).

Furthermore, the interaction between period and different covariates was assessed using a GEE model to determine whether the CPR differed within certain categories of these covariates.

## Results

A total of 606 women received 1995 IUI cycles with frozen donor semen. Outcome results were unavailable for 12 cycles (0.6%). The CPR per cycle was 18.2 % (361/1983). Per pregnancy multiple pregnancy rate in our study was 5.5 % (20/361).

### Univariate GEE analysis

Univariate statistical analyses showed that the CPR increased from 16.6 % (period 1) to 20.8 % (period 2), a significant difference (p=0.01).

The CPR per cycle significantly differed with patient age (p = 0.0002). Also, patients presenting with primary infertility showed a significantly lower pregnancy rate compared with patients suffering from secondary infertility (P = 0.0039). Smoking and women’s BMI were not associated with CPR (Table I).

**Table I:** Univariate analysis of patient-related characteristics and specific covariates. BMI = body mass index; CP = clinical pregnancy (number); CPR = clinical pregnancy rate (%); SE = standard error. Significant p-values indicate a statistical difference between CPRs among the different categories. Multivariable analyses will determine which categories significantly differ from each other.

Parameter	CP	Total	CPR	SE	p-value
Age (years)					**.0002**
< 30	83	449	18.5	1.8	
30-34.99	164	811	20.2	1.4	
35-39.99	103	551	18.7	1.7	
≥40	11	170	6.5	1.9	
Infertility					**.0039**
Primary	234	1427	16.4	1.0	
Secondary	127	554	22.9	1.8	
Smoking					.3451
No	317	1780	17.8	0.9	
Yes	40	190	21.1	2.9	
BMI (kg/m^2^)					.8361
< 20	26	149	17.5	3.1	
20-24.99	177	961	18.4	1.3	
25-29.99	91	488	18.7	1.8	
≥30	66	379	17.4	2.0	
Stimulation					**.0004**
NC	167	985	17.0	1.2	
CC	78	530	14.7	1.5	
HMG/rFSH	114	464	24.6	2.0	
HCG-insemination interval (h)					.1526
<15	28	171	16.4	2.8	
15−22.99	126	780	16.2	1.3	
≥23	207	1032	20.1	1.3	
Oestradiol day 0 (ng/l)					.9578
1.69−194	87	484	18.0	1.8	
195−267	86	452	19.0	1.9	
268−414	89	483	18.4	1.8	
415−1976	72	410	17.6	1.9	
Progesterone day 0 (µg/l)					.0751
<0.5	190	995	19.1	1.3	
0.5−0.99	144	780	18.5	1.4	
1−1.49	23	156	14.7	2.8	
≥1.5	4	52	7.7	3.7	

Stimulation method was significantly associated with CPR (p = 0.0004). The time interval between HCG triggering and insemination as well as the hormone levels of oestradiol and progesterone on day 0 of the cycle did not show a significant association with pregnancy rate (Table I). None of the post-thaw sperm quality parameters showed a significant influence on CPR (Table II).

**Table II:** Univariate analysis of post-thaw sperm-quality factors. CP = clinical pregnancy; CPR = clinical pregnancy rate (with foetal heartbeat); IMC = inseminating motile count; TMSC = total motile sperm count. All post-thaw sperm parameters were categorized for analysis, none of the results was significantly different.

Parameter	CP	Total	CPR	SE	p-value
IMC (million)					.1886
< 5	93	574	0.162	0.015	
5-9.99	147	764	0.192	0.014	
≥10	121	645	0.188	0.015	
Concentration (million/ml)					.5368
0.1−49	33	228	0.145	0.023	
50-99	249	1362	0.186	0.011	
100-149	69	343	0.201	0.022	
150-340	10	50	0.200	0.057	
Grade A motility (%)					.5128
0−9	26	172	0.151	0.027	
10-19	101	595	0.170	0.015	
20-29	124	657	0.189	0.015	
30-39	84	407	0.206	0.020	
≥40	26	152	0.171	0.031	
Progressive motility (%)					**.7323**
5-39	56	313	0.179	0.022	
40-54	154	862	0.179	0.013	
55-69	137	710	0.193	0.015	
70-85	14	98	0.143	0.035	

### Multivariable GEE analysis

A summary of the results of the univariate analyses is shown in Table III. Only the significant covariates (p<0.20, in bold) were taken into account when building the final multivariable GEE model. The final multivariable model indicates that only female age, ovarian stimulation, and primary/ secondary infertility significantly influence CPRs (Table IV). Women aged less than 40 had a significantly higher pregnancy rate compared to women over 40. Women with primary infertility had a lower pregnancy rate compared to secondary infertility. Pregnancy rates were lower for NC and CC compared to hMG/rec FSH.

**Table III:** IUI donor semen: Summary of the univariateIUI donor semen: Summary of the univariate analyses. Only the significant covariates (p<0.20, in bold) are taking into account for the multivariate GEE model (IMC =Inseminating Motile Count after washing procedure, TMSC = Total Motile Sperm Count before washing).

Parameter	p-value
**Age patient**	**.0002**
Smoking patient	.3451
BMI patient	.8361
**Infertility (primary or secondary)**	**.0039**
Attempt (rank)	.4234
**Ovarian stimulation**	**.0004**
**HCG-insemination interval**	**.1526**
Oestradiol D0 (ng/l)	.9578
**Progesterone D0 (µg/l)**	**.0751**
Blood loss	.9017
**IMC (million)**	**.1886**
Sperm concentration (million/ml)	.5368
Sperm grade A motility (%)	.5128
Sperm progressive motility (%)	.7323
TMSC (million)	.8653
Sperm washing method	.5808
**Slow-Release Insemination**	**.0176**

**Table IV:** Results from the multivariable generalized estimating equations analysis IUI donor semen. (NC = natural cycle, CC = clomiphene citrate, hMG = human Menopausal Gonadotrophins, rec FSH = recombinant FSH, SR-IUI = slow-release intrauterine insemination).

Covariate	Parameter estimation (SE)	p-value
SRI-IUI		.0614
Age partner		<.0001
< 30 vs 30 - 34.99	-0.0216 (0.1519)	.8869
< 30 vs 35 - 39.99	0.1330 (0.1672)	.4263
**< 30 vs ≥40**	1.4010 (0.3454)	**<.0001**
30 - 34.9 vs 35-<40	0.1546 (0.1447)	.2852
**30 - 34.99 vs ≥40**	1.4226 (0.3339)	**<.0001**
**35 - 39.99 vs ≥40**	1.2680 (0.3415)	**.0002**
Prim/Sec infertility	-.3794 (0.1362)	.0078
Ovarian stimulation		.0004
NC vs CC	0.16261 (0.1448)	.2935
**NC vs hMG/rec FSH**	-0.5338 (0.1437)	**.0002**
**CC vs hMG/rec FSH**	-0.6964 (0.1663)	**<.0001**

Although the CPR increased from 16.6 % (period 1) to 20.8 % (period 2), this difference was not significant anymore (p=0.06) when accounting for other covariates (Table IV).

### Outcome results period 1 (bolus IUI) versus period 2 (slow-release IUI)

The CPR per cycle was 16.6 % (208/1248) in period 1 compared to 20.8 % (153/735) in period 2, a non-significant increase (p=0.06). The multiple pregnancy rate in this cohort was 8.1% (17/208) in period 1 and 1.9 % (3/153) in period 2 (p = 0.01). The main characteristics of the study population in both periods are shown in Table V. In period 2 we found a significantly higher female age. a longer HCG-IUI time interval and a lower grade A motility and progressive motility (Table V).

**Table V:** IUI donor semen: Main characteristics of the study population before 2016 and after 2015. Data are presented as mean +/- SD (bold = statistically significant difference p < 0.05).

Parameter	Before 2016	After 2015	p-value
Patient characteristics			
Age patient (years)	32.6 +/- 0.2	34.6 +/- 0.2	**<.0001**
BMI patient (kg/m^3^)	25.6 +/- 0.2	26.0 +/- 0.3	.1115
IUI procedure characteristics			
HCG-insemination intervval (h)	21.1 +/- 0.2	25.6 +/- 0.1	**<.0001**
Sperm characteristics			
IMC (million)	8.5 +/- 0.2	8.6 +/- 0.3	.7796
Concentration (million/ml)	80.0 +/- 1.1	81.0 +/- 1.3	.5150
**Grade A motility** (%)	24.7 +/- 0.4	21.4 +/- 0.4	**<.0001**
**Progressive motility** (%)	53.4 +/- 0.4	47.8 +/- 0.4	**<.0001**

## Discussion

The request for donor insemination increased significantly in Belgium (Pennings, 2004; Pennings et al., 2009; Thijssen et al., 2014). To be able to accurately predict the likelihood of success after IUI treatment with donor semen remains an important challenge. In this study, the relationship between certain covariates, such as female age, smoking habits, BMI, use or non-use of ovarian stimulation, post-thaw semen characteristics and pregnancy outcome was explored through a prospective analysis of 1995 cycles in a total of 606 women presenting at a tertiary referral infertility centre between July 2011 and December 2018. Data were registered through CRFs completed by the midwife together with the patient. Statistical analysis was conducted using GEE to account for the correlation between observations from the same patient.

Our results showed an overall CPR per cycle of 18.2%, which was higher than the average CPR of 13.9 % reported in the latest 2017/2018 data of the Belgian Register for Assisted Procreation (www.belrap.be). Although the overall CPR in our study group was generally high, the multiple pregnancy rate (MPR) was only 5.5 %, a low figure when compared to the European 2016 data (Wyns et al., 2020).

Our low MPR can be explained by a strict protocol in which the insemination was cancelled when three or more follicles of 11 mm or wider were observed. Furthermore, ovarian stimulation with hMG or rec FSH was only used in 23.4 % of cycles. Clomiphene citrate stimulation and natural cycle IUI was performed in respectively 26.8 % and 49.8 % of cases. The MPR was 10.7% in cycles stimulated with HMG and/or recombinant FSH, 8.8% in cycles stimulated with clomiphene citrate and 1.2% in NC.

Of all examined covariates possibly influencing clinical pregnancy rates, the multivariable GEE model indicated a significant influence on CPR for only 3 parameters:

Women presenting with secondary infertility appeared to have higher CPRs compared with primary infertile women. Secondary infertile women have been pregnant before (either normal or pathological).

Female age is the most relevant predictor of the probability of clinical pregnancy in IUI treatment and evidence-based data show that a sharp decline of IUI success rate is observed in women over the age of 40 years, which is presumably related to oocyte quality. Our results also showed a significantly lower CPR in patients aged 40 years or older, confirming the results of many previous reports (Botchan et al., 2001; De Brucker et al., 2009; Ferrara et al., 2002; Zuzuarregui et al., 2004).

Results from our multivariable GEE analysis indicated that cycles stimulated with HMG and recombinant FSH resulted in significantly higher pregnancy rates compared with cycles stimulated with clomiphene citrate or NC as shown in Table III. These results are largely in accordance with previous reports showing significantly higher pregnancy rates after donor IUI treatment using recFSH or HMG, and the latter higher than clomiphene citrate stimulated cycles (Matorras et al.,2002; Zuzuarregui et al., 2004). On the other hand, Ferrara et al. (2002), Botchan et al. (2001) and De Brucker et al. (2009) were not able to show a significant influence of using different ovarian stimulation protocols on pregnancy and delivery rates resulting from IUI with donor semen.

Post-thaw IMC and other sperm factors did not show a significant influence on CPR in our study confirming the results of a prospective randomized trial reported by Rodriguez-Purata et al. (2018). Smoking did not affect the CPR in our study. Huyghe et al. (2017) showed that female non-smoking or smoking less than 15 cigarettes daily turned out to be significantly associated with a higher clinical pregnancy rate after IUI-D compared to women smoking more than 15 cigarettes daily. Farhi and Orvieto (2009), however, were unable to detect a significant difference in pregnancy rates between groups of smoking and non-smoking women in a study on the influence of smoking on the outcome of homologous IUI with ovarian stimulation.

Studies on methods aiming to increase the pregnancy rate after donor inseminations are scarce. Many studies on homologous IUI have investigated different strategies to increase the pregnancy rate. Most of these studies deal with ovarian stimulation protocols, timing of IUI, sperm quality factors, sperm processing techniques etc. Only a limited number of studies have dealt with the technique itself.

The slow-release insemination (SRI) instead of the regular bolus IUI injection turned out to be a possible strategy to increase the pregnancy rate after IUI. In a randomized cross-over study with a Grasby type MS16 pump for 3 hours Muharib et al. (1992) reported an improvement in clinical pregnancy rate per cycle and cumulative pregnancy rate after 4 cycles from 6.1% to 22% and 15.0% to 63.1% respectively. According to the authors this can be explained by increasing the period of potential fertilization through injecting of a persistent low concentration of spermatozoa.

As a result of two pilot randomized, controlled cross-over studies Marschalek et al. (2017) found a statistically significant advantage of SRI over conventional bolus IUI. The EVIE device was used for slow-release injection in this study. The same authors recently reported the results of a multicentre, randomized cross-over trial comparing bolus IUI with SRI, using the same EVIE device (Marschalek et al., 2020). Only in a subgroup of women aged under 35 years, the pregnancy rate with SRI was significantly better compared to bolus IUI (17% versus 7%, relative risk 2.33; p = 0.032).

We recently published the outcome results of performing SRI (period 1) versus bolus-IUI (period 2) in a large series of 2565 homologous inseminations, making use of the data of the same prospective cohort study as the one we used in this study (Ombelet et al., 2021). For homologous inseminations we found a significant 4.5 % increase in CPR from 9.0 to 13.5 % per cycle in period 2. In this study, and although the CPR also increased from 16.6 to 20.8 % (4.2 %) in period 2, this difference was not statistically significant after accounting for other covariates in the multivariable GEE analysis (p=0.06). Nevertheless, a trend towards a better success rate (CPR) after SRI-IUI versus bolus-IUI can also be seen in our donor insemination programme, the difference was significant after univariate analysis but couldn’t be confirmed after GEE analysis.

Comparing the patients’ characteristics in period 2 versus period 1, we found a higher female age, a lower grade A motility and a lower progressive motility (Table IV), just the opposite of what one would expect considering the higher CPR in period 2.

Though the data was collected prospectively, comparisons are made between data collected at different points in time, a weakness of this study. For example, the HCG-IUI time interval was significantly longer in period 2 caused by a re- organization in our IUI programme. On the other hand, in a Cochrane review it was concluded that comparable results can be achieved when IUI is performed between 12 to 36 hours after HCG injection (Cantineau et al., 2014). Therefore, we assume that this difference in timing between both groups is not important although it should be addressed when planning a prospective randomized trial.

This study provides some important strengths. First, collection of the data was prospective, as different patient and treatment-specific factors were recorded by means of a CRF at the time of insemination. The results of the CRFs were examined by a third person for possible lack of data monthly. Second, the multivariable GEE analysis used in this study has a major advantage over previously used ordinary logistic regression models as it takes into account the correlation between observations from the same patient when patients are coming back for treatment after previous failed attempts. Last but not least, only one study aspect changed in period 2 compared to period 1, although this parameter was twofold: slow-release IUI and a more patient- centred approach.

## Conclusions

The results of our prospective cohort study investigating the influence of modified slow- release IUI versus bolus-IUI showed a non- significant increase in CPR of 4.2 % after using the generalized estimating equations (GEEs) for statistical analysis.

After careful power analysis future prospective randomized studies have to be performed to investigate whether SRI or patient-friendly measurements or both may lead to an increased pregnancy rate after donor insemination.

## References

[1] Botchan A, Hauser R, Gamzu R (2001). Results of 6139 artificial insemination cycles with donor spermatozoa.. Hum Reprod.

[2] Cantineau AE, Janssen MJ, Cohlen BJ (2014). Synchronised approach for intrauterine insemination in subfertile couples.. Cochrane Database Syst Rev.

[3] De Brucker M, Haentjens P, Evenepoel J (2009). Cumulative delivery rates in different age groups after artificial insemination with donor sperm.. Hum Reprod.

[4] Depuydt CE, Verstraete L, Berth M (2016). Human Papillomavirus Positivity in Women Undergoing Intrauterine Insemination Has a Negative Effect on Pregnancy Rates.. Gynecol Obstet Invest.

[5] Depuydt CE, Donders G, Verstraete L (2018). Time has come to include Human Papillomavirus (HPV) testing in sperm donor banks.. Facts Views Vis Obgyn.

[6] Depuydt CE, Donders GGG, Verstraete L (2019). Infectious human papillomavirus virions in semen reduce clinical pregnancy rates in women undergoing intrauterine insemination.. Fertil Steril.

[7] Farhi J, Orvieto R (2009). Influence of smoking on outcome of COH and IUI in subfertile couples.. J Assist Reprod Genet.

[8] Ferrara I, Balet R, Grudzinskas JG (2002). Intrauterine insemination with frozen donor sperm. Pregnancy outcome in relation to age and ovarian stimulation regime. Hum Reprod.

[9] Huyghe S, Verest A, Thijssen A (2017). Influence of BMI and smoking on IUI outcome with partner and donor sperm.. Facts Views Vis Obgyn.

[10] Matorras R, Diaz T, Corcostegui B (2002). Ovarian stimulation in intrauterine insemination with donor sperm: a randomized study comparing clomiphene citrate in fixed protocol versus highly purified urinary FSH.. Hum Reprod.

[11] Molenberghs G Verbeke G Models for discrete longitudinal data. Springer Series in Statistics; Springer, New-York: 2005

[12] Marschalek J, Franz M, Gonen Y (2017). The effect of slow release insemination on pregnancy rates: report of two randomized controlled pilot studies and meta-analysis.. Arch Gynecol Obstet.

[13] Marschalek J, Egarter C, Vytiska-Binsdorfer E (2020). Pregnancy rates after slow-release insemination (SRI) and standard bolus intrauterine insemination (IUI) - A multicentre randomised, controlled trial.. Sci Rep.

[14] Muharib NS, Abdel Gadir A, Shaw RW (1992). Slow release intrauterine insemination versus the bolus technique in the treatment of women with cervical mucus hostility.. Hum Reprod.

[15] Ombelet W, Van der Auwera I, Bijnens H (2021). Improving IUI success by performing modified slow-release insemination and a patient-centred approach in an insemination programme with homologous semen: a prospective cohort study.. Facts Views Vis Obgyn.

[16] Pennings G (2004). Legal harmonization and reproductive tourism in Europe.. Hum Reprod.

[17] Pennings G, Autin C, Decleer W (2009). Cross-border reproductive care in Belgium.. Hum Reprod.

[18] Rodriguez-Purata J, Latre L, Ballester M (2018). Clinical success of IUI cycles with donor sperm is not affected by total inseminated volume: a RCT.. Hum Reprod Open.

[19] Thijssen A, Dhont N, Vandormael E (2014). Artificial insemination with donor sperm (AID): heterogeneity in sperm banking facilities in a single country (Belgium).. Facts Views Vis Obgyn.

[20] Thijssen A, Creemers A, Van der Elst W (2017a). Predictive value of different covariates influencing pregnancy rate following intrauterine insemination with homologous semen: a prospective cohort study.. Reprod Biomed Online.

[21] Thijssen A, Creemers A, Van der Elst W (2017b). Predictive factors influencing pregnancy rates after intrauterine insemination with frozen donor semen: a prospective cohort study.. Reprod Biomed Online.

[22] Van Hoof W, Pennings G, De Sutter P (2015). Cross-border reproductive care for law evasion: a qualitative study into the experiences and moral perspectives of French women who go to Belgium for treatment with donor sperm.. Soc Sci Med.

[23] Wyns C, Bergh C, Calhaz-Jorge C (2020). ART in Europe, 2016: results generated from European registries by ESHRE. Hum Reprod Open.

[24] Zuzuarregui JL, Meseguer M, Garrido N (2004). Parameters affecting the results in a program of artificial insemination with donor sperm. A 12-year retrospective review of more than 1800 cycles. J Assist Reprod Genet.

[25] Zeger SL, Liang KY (1986). Longitudinal data analysis for discrete and continuous outcomes.. Biometrics.

